# Dataset on hydraulic burst pressure test of Type IV composite pressure vessel

**DOI:** 10.1016/j.dib.2025.111333

**Published:** 2025-01-27

**Authors:** Caroline Lüders, Sven Ropte, Daniel Schmidt, Martin Liebisch

**Affiliations:** Institute of Lightweight Systems, German Aerospace Center, Lilienthalplatz 7, 38108 Braunschweig, Germany

**Keywords:** Composite pressure vessel, Type IV hydrogen vessel, Burst pressure test

## Abstract

The present dataset belongs to a hydraulic burst pressure test of one Type IV vessel designed to burst at 200 bar. Before testing, the vessel was inspected by ultrasonic measurements. During burst pressure test, strain gauges at nine positions within the cylindrical part and on one dome of the tank recorded the deformation behavior of the vessel. The dataset provides information on the nominal tank design and winding layup, data of geometrical measurement of a nominal identical vessel, data of the ultrasonic inspection and the pressure and strain gauge data recorded during burst pressure test. Assembling this information, the dataset provides an experimental validation basis for simulation methods aiming to predict deformation and damage behavior of composite pressure vessels.

Specifications Table

**Table:** 

Subject	Mechanical Engineering
Specific subject area	Burst pressure test of composite pressure vessels
Type of data	Figures (.png, .bmp) Time series data (.csv) Geometric data (.ginspect, .stl)
	Tabular data (.csv, .xlsx) ASCII text data (.txt) Raw data and processed data
Data collection	One Type IV vessel was pressurized until burst failure. The internal pressure was applied by a hydraulic high-pressure pump using water as test fluid and measured by a pressure sensor. Strain gauges recorded the strains in hoop and in axial direction at nine positions on the tank. Before testing, the cylindrical regime of the vessel was examined by ultrasonic scanning. The outer surface geometry of another nominally identical vessel and its liner was measured by optical 3D scanning technique and the outer liner contour line was reconstructed using the software ZEISS INSPECT Optical 3D. Cut outs were extracted from this vessel and thickness measurements were performed using a micrometer gauge.
Data source location	Optical 3D scanning, ultrasonic scanning and instrumentation of the vessel with strain gauges, extracting the cut outs and thickness measurement were performed at the Institute of Lightweight Systems of the German Aerospace Centre in Braunschweig, Germany. The burst pressure test was performed in the Harz Mountains, Germany. All data collected before and during the burst pressure test are stored on the servers of the Institute of Lightweight Systems in Braunschweig, Germany.
Data accessibility	Repository name: zenodo Data identification number: 10.5281/zenodo.10608733 Direct URL to data: https://zenodo.org/records/10983652

## 1 Value of the Data

• This dataset provides data on the mechanical behavior (local strains and burst pressure) of a Type IV composite pressure vessel subjected to internal pressure load. In addition to the measurement data obtained during the test, the dataset and the presented paper provide comprehensive information on the vessel geometry, the composite material and the winding layup. This makes the dataset valuable for the following purposes.
• The development of new simulation methods for predicting mechanical behavior and burst pressure of composite vessels always requires experimental validation data for model evaluation. Thus, researchers and engineers in this field may benefit from the presented dataset as it provides such an experimental validation data basis. The information provided about the vessel geometry, layup and material may serve as input data for the simulation models. By comparing the model prediction regarding local strains and burst pressure with the corresponding provided test data, model developers are able to evaluate their models’ quality.
• Researchers and engineers might use the dataset for comparison purposes. Test data obtained for other composite pressure vessel designs can be compared to the provided data. Thus, the dataset facilitates a comparison of the mechanical performance of different vessel designs.
• The dataset supports the research on and the development of hydrogen pressure vessels which is of increasing importance for various transportation modes (rail way, automotive, aviation).

## 2 Background

Addressing the global goal to replace fossil fuels, hydrogen is handled as alternative fuel for automotive, railway and aviation applications. Composite pressure vessels of Type IV compose of a plastic liner and a winding made of carbon fiber reinforced plastics (CFRP) and are currently used in automotive industry and also demonstrated for railway applications. However, there is still a need to optimize the ratio of the vessel's structural mass to the stored hydrogen mass. The provided dataset was obtained within research activities in the EU funded project Rail4Earth aiming on the development of optimization methods for lightweight pressure vessels and of simulation models to numerically predict burst pressure and fatigue life of such vessels. Within this project one vessel manufactured according to the optimized design was assessed by a burst pressure test. These test results are provided within the presented dataset.

## 3 Data Description

The dataset described within this article provides test results of a burst pressure test of one Type IV composite vessel. This also contains geometric information on the vessel, data of the pre-test ultrasonic inspection as well as pressure and strain gauge data obtained during the burst test. The referenced dataset contains the folders and files listed in Table 1. The tested vessel is one of a batch of three nominal identical composite vessels labelled SN01, SN02 and SN03. Files referring to the tested vessel SN03 contains “SN03” in the file name. Some geometric information is only available for the vessel SN01 and files containing this information are named accordingly (“SN01”). Files not assigned to one specific sample are valid for all vessels. The files containing “raw” in the file name represent the pure data measured. The data in other files are processed or derived from the raw data as described below.

**Table 1:** List of folders and files within the dataset.

Data file name	Content
Readme.txt	summarized information on the provided dataset

Vessel_data.7z	Zipped folder containing information on the vessel
Vessel_sketch.png	sketch of the vessel explaining geometric parameters (Fig. 1)
3D_scanning_liner_raw_SN01.ginspect	data of optical 3D scanning of the liner of vessel SN01 in original format of the used measurement system ATOS*
3D_scanning_liner_raw_SN01.stl	data of optical 3D scanning of the liner of vessel SN01 in platform agnostic stl-format
3D_scanning_vessel_raw_SN01.ginspect	data of optical 3D scanning of the vessel SN01 in original format of the used measurement system ATOS*
3D_scanning_vessel_raw_SN01.stl	data of optical 3D scanning of the vessel SN01 in platform agnostic stl-format
Outline_contour_liner_ SN01.txt	half outline contour of liner of vessel SN01 reconstructed from optical 3D scanning results; given by axial and radial coordinates in the coordinate system depicted in Winding_parameters.png (Fig. 3)
Winding_parameters.png	sketch of the winding explaining winding parameters (Fig. 3)
layupbook.txt	table describing the parameters of the CFRP winding according to Winding_parameters.png (Fig. 3)
SN01_cutouts_thickness_measurement.xlsx	Excel file with tabular data of thickness measurement for two cut outs extracted from vessel SN01.

Ultrasonic_data.7z	Zipped folder containing data regarding the ultrasonic scanning
testsetup_US.png	sketch of the test setup for the ultrasonic inspection (Fig. 6)
US_coordinate_system.png	sketch showing the ultrasonic scan coordinate system with respect to the vessel geometry and to the strain gauge positions (Fig. 7)
ultrasonic_flaw_echo_CScan_SN03.bmp	C-Scan of the flaw echo generated from the ultrasonic inspection of vessel SN03
ultrasonic_back_wall_echo_CScan_SN03.bmp	C-Scan of the back-wall echo generated from the ultrasonic inspection of vessel SN03
ultrasonic_DScan_SN03.bmp	D-Scan (flaw depth) generated from the ultrasonic inspection of vessel SN03

Burst_test_data.7z	Zipped folder containing data regarding the burst test
sensor_position_strain_gauges.png	sketch of the strain gauge positions on the vessel (Fig. 9)
pressure_raw_SN03.csv	raw data of the pressure signal over time; sample rate 100Hz
sensorinfo_straingauges_SN03.csv	Tabular data providing the strain gauge sensor locations, its measuring direction and the sensor meta data; sensor locations (x,φ) are given with respect to the coordinate system shown in sensor_position_strain_gauges.png (Fig. 9); measuring direction is axial (A) along the tank axis or circumferential (U)
straingauges_raw_SN03.csv	raw strain gauge data over time; sample rate 300Hz
straingauges_and_pressure_10hz_SN03.csv	combination of “straingauges_raw_SN03.csv” and “pressure_raw_SN03.csv” whereas pressure data and strain gauge data are synchronized with respect to time; sample rate reduced to 10 Hz
straingauges_and_pressure_10hz_zeroized_SN03.csv	representation of “straingauges_and_pressure_10hz_SN03.csv”’ with zeroized starting point of the strain gauge data (subtraction of the values at time zero); sample rate 10Hz

*to be opened with software ZEISS INSPECT Optical 3D.

The strain gauge names in the data files follow the structure: SG_<Position No.>_<orientation>. The position number reaches from “1” to “9” (positions are given in Fig. 9). The orientation of the strain gauges is “U” for circumferential direction or “A” for axial direction. An example: SG_01_A is the strain gauge applied at position 01 measuring the strains in axial direction, whereas SG_01_U is located at the same position but measuring the hoop strains. The strain gauge signals are given as strain along the measuring direction of the corresponding strain gauge in µm/m. The time in the time series data is given in seconds and the pressure is given in bar.

The pressure and strain gauge data were measured in individual data acquisition systems during the burst test. They are given in “pressure_raw.csv” and “straingauges_raw.csv” with each file referring to its own time data. The method for time synchronization of strain gauge and pressure data provided in “straingauges_and_pressure.csv” is described in the section “Burst pressure test”.

## 4 Experimental Design, Materials and Methods

### 4.1 Description of the composite vessel

**Fig. 1:**
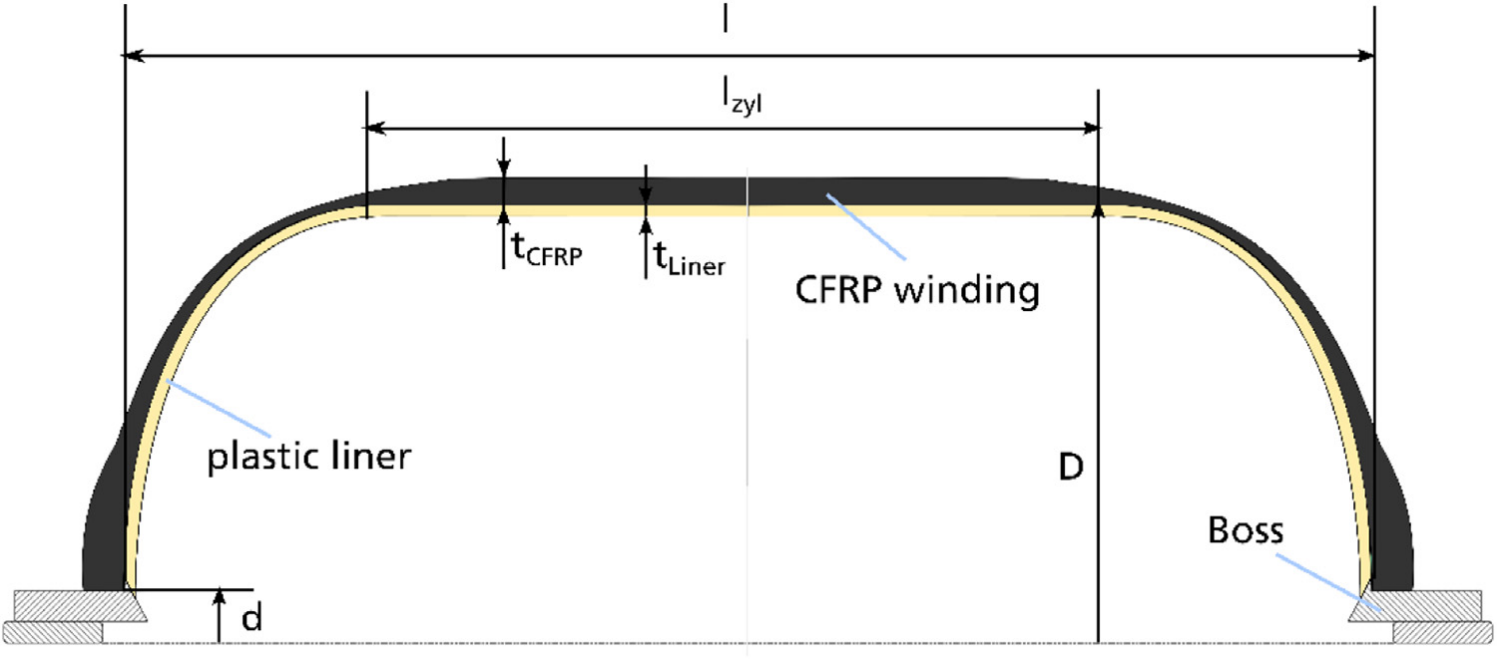
Vessel geometry.

The tested vessel SN03 is one of a batch of three nominal identical composite vessels manufactured by wet winding process at INVENT GmbH in Braunschweig, Germany. The vessel geometry is sketched in Fig. 1.

**Fig. 2:**
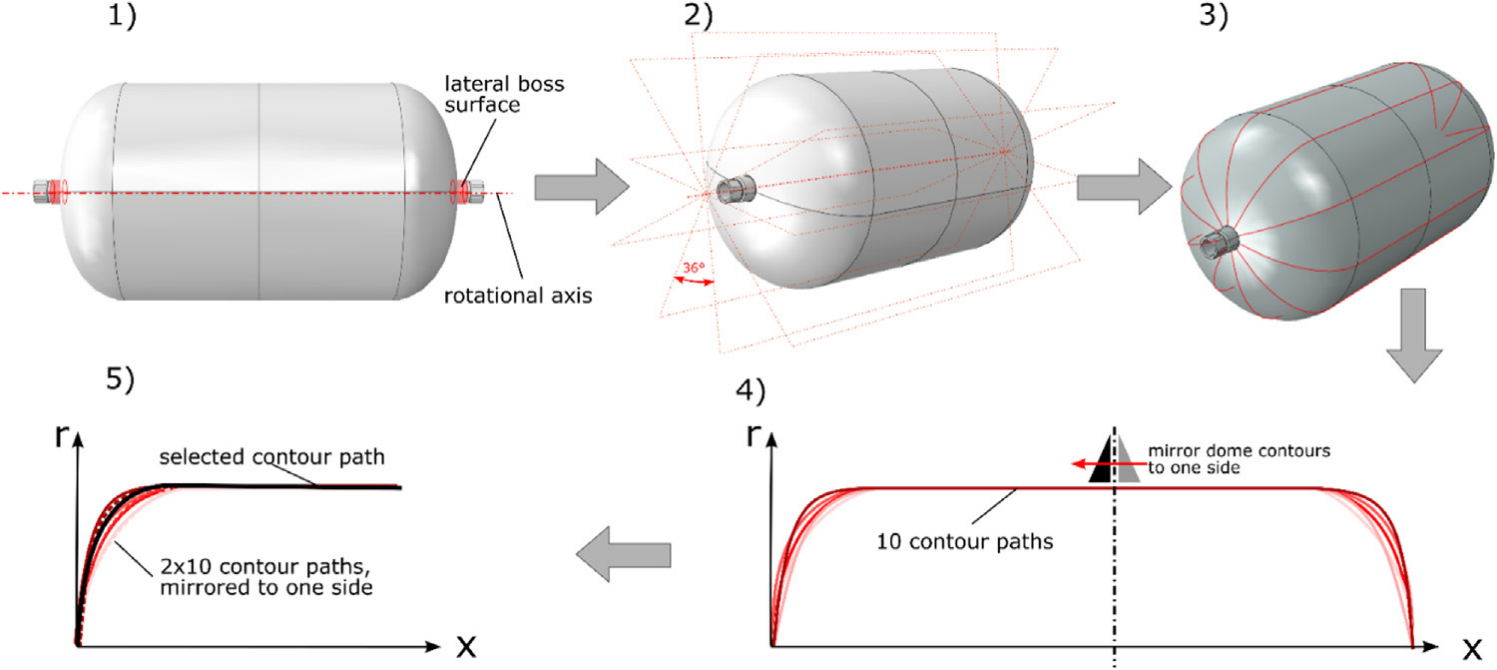
Process of liner contour reconstruction based on optical 3D scanning data: (1) define axis of rotation using the lateral boss surfaces; (2) create ten planes in angular distance of 36°; (3) get ten contour lines from intersection of the planes with measured liner surface; (4) rotate all contour lines on one plane and plot in x-r-Diagram (x – axial tank coordinate, r – radial coordinate); (5) mirror all contour lines to one dome side and choose contour line with smallest deviation to all other contour paths.

Plastic liners made of PA12 were used as tooling for the wet winding process and remained in the tank to ensure the tank's tightness. They were manufactured by a rotational moulding process and are nominal identical for all vessels of the batch. The liner of vessel SN01 was inspected by structured-light 3D scanning technique using the measuring system ATOS by Carl Zeiss GOM Metrology GmbH. This technique enables a 3D scan of the outer liner surface. Fig. 2 visualizes the procedure to reconstruct a liner contour usable for generating the filament winding paths from the 3D scanning data of the liner (3D_scanning_liner_raw_SN01.ginspect). In the software ZEISS INSPECT Optical 3D the rotational axis was first created based on the lateral surfaces of the visible cylindrical bosses (1). Based on this axis, ten section planes with the same angular distance (36°) were created through the measured surface (2). The intersections of the measured surface and the section planes yield ten contour paths of the liner (3). The section planes, the rotational axis and the contour paths are stored in the provided 3D scanning data file (3D_scanning_liner_raw_SN01.ginspect). For contour reconstruction, they were imported into the 3D CAD program Catia V5 and the contour paths were all rotated onto one plane (4), halved and mirrored to one dome side (5). From the 20 paths now available, the path with the smallest deviation from all other paths was selected (5). Points were then created on this path and a curvature-continuous spline was developed based on these points. On this spline, points were created to export the contour data which are provided in “Outline_contour_liner_ SN01.txt” in “Vessel_data.7z”. The liner dimensions listed in Table 2 were determined based on this idealized contour.

**Table 2:** Liner dimensions of vessel SN01.

Symbol	Parameter	Unit	Value
D	Outer liner diameter	mm	412.41
d	Polar opening diameter	mm	46
l_zyl_	Length of cylindrical regime	mm	534
l	Whole liner length	mm	770.72
t_Liner_	Liner thickness	mm	8

The CFRP of the winding consists of the fibre T700SC-12,000-60E-P1-12k (grammage $\rho_{A}$= 800 tex = 800 g/km and density $\rho_{F}$=1,8 g/cm³) and the matrix system LY556/HY917-1CH/DY070. Material parameters for that material taken from [1] are listed in Table 3.

**Table 3:** Elasticity and strength parameters of T700SC/LY556 taken from [1].

Symbol	Parameter	Unit	Value
$E_{11}^{t}$	Young's modulus in fibre direction for tension	GPa	129.4
$E_{11}^{c}$	Young's modulus in fibre direction for compression	GPa	110.7
$E_{22}^{t}$	Young's modulus transverse fibre direction for tension	GPa	8.05
$E_{22}^{c}$	Young's modulus transverse fibre direction for compression	GPa	8.87
*G*_12_	In-plane shear modulus	GPa	3.91
*G*_23_	Out-of-plane shear modulus	GPa	2.85
$\upsilon_{12}$	In-plane Poisson's ratio	–	0.317
$\upsilon_{23}$	Out-of-plane Poisson's ratio	–	0.41
$R_{11}^{t}$	Tensile strength in fibre direction	MPa	2089
$R_{11}^{c}$	Compression strength in fibre direction	MPa	1032
$R_{22}^{t}$	Tensile strength transverse fibre direction	MPa	36.2
$R_{22}^{c}$	Compression strength transverse fibre direction	MPa	164.4
$R_{12}$	In-plane shear strength	MPa	52.2

**Fig. 3:**
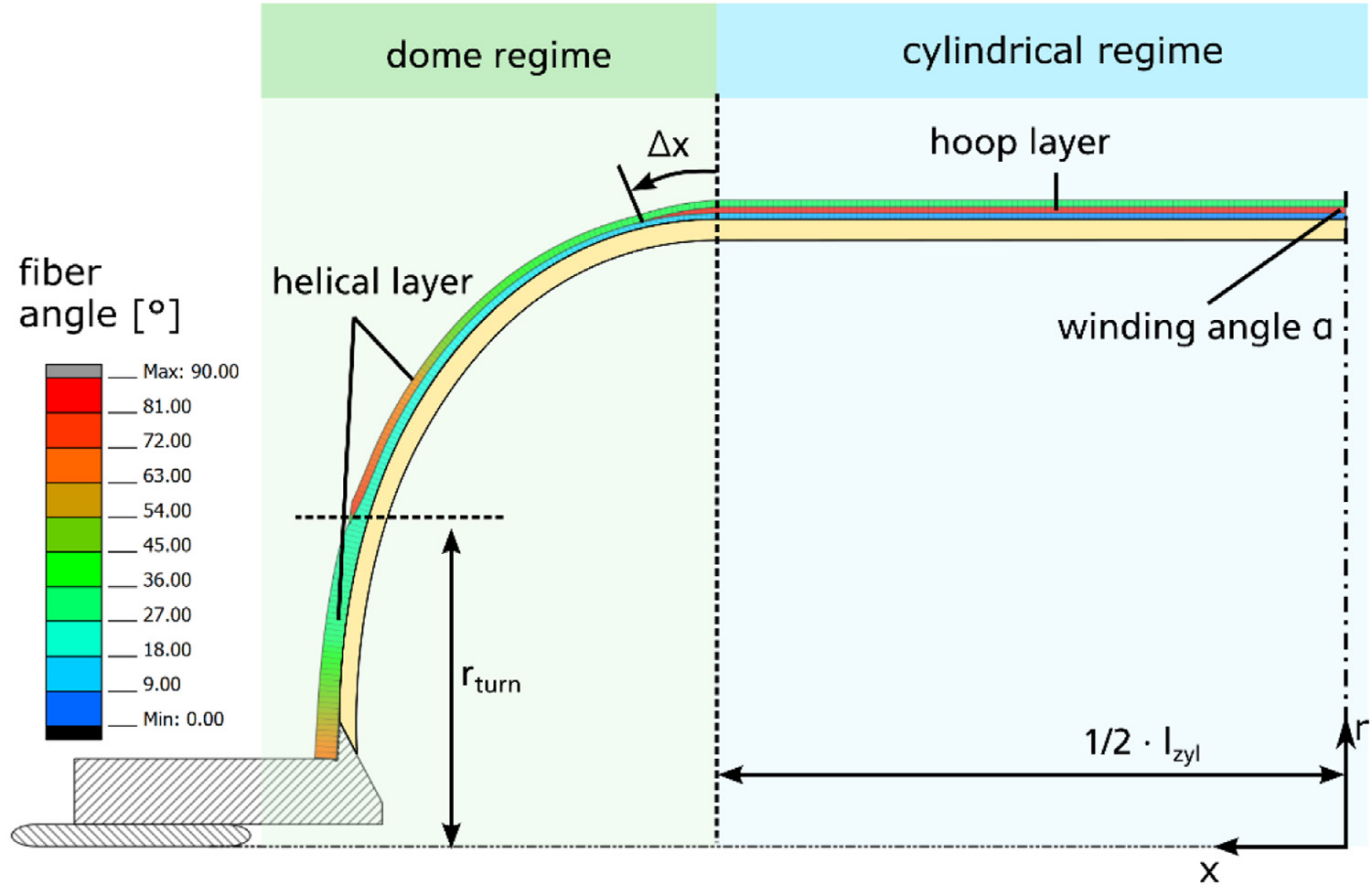
Winding parameters.

The winding laminate has been designed to withstand 200 bar burst pressure. Table 4 lists the parameters of the nominal winding laminate which are explained by Fig. 3.

**Table 4:** Parameters of nominal winding layup.

Layer No.	Layer type	Winding angle α [°] in cylindrical regime	Hoop shift Δx [mm]	Turning radius r_turn_ [mm]	Wound layer thickness [mm]
1	helical	8.6	–	23.0	0.474
2	hoop	90.0	15.0	205.08	0.452
3	helical	8.6	–	23.001	0.474
4	hoop	90.0	5.0	207.7	0.452
5	helical	8.6	–	23.002	0.474
6	hoop	90.0	−5.0	208.52	0.452
7	helical	22.23	–	72.87	0.474

nominal layup thickness in cylindrical regime:					3.25

A symmetrical realization of the wound laminate by the wet winding process is assumed, resulting in the same values of the layer hoop shifts and of the turning radii for both domes. According to the information given by the manufacturer, helical layers are wound with a tow width of *w_tow_*=3.125 mm. Due to the higher contact pressure, the tow width appears to be *w_tow_*=3.275 mm for hoop layers. In the wet winding process four tows are used forming a band width of *b_w_* = 12.5 mm for helical and *b_w_* = 13.1 mm for hoop layers. As shown in Eq. (1), the tow thickness *t_tow_* is related to the tow width via the fiber grammage $\rho_{A}$, fiber density $\rho_{F}\;$and the fiber volume fraction (*FVC*) of the tow. In Eq. (1), *A_rov_* is the cross section of the dry fiber roving before impregnating with resin.

(1) $$t_{tow}=\;\frac{A_{rov}}{w_{tow}\cdot FVC}=\frac{\rho_{A}}{\rho_{F}}\cdot \frac{1}{FVC\;\cdot \;w_{tow}}$$

To reach an *FVC* of around 60 %, it was aimed for a nominal tow thicknesses for helical layers of 0.237 mm and for hoop layers of 0.226 mm. Due to the band trajectories in the winding process, the band covers each point of the mandrel twice. Thus, the resulting thickness of a wound layer in the cylindrical region of the tank is twice the tow thickness. The nominal values of the wound layer thicknesses are given in Table 4. Summing up, the nominal thickness of the CFRP winding reaches 3.25 mm in the cylindrical region. The transition layers, used during the winding process to change the winding angle between one layer to the next one, are not considered in the layup definition and in the thickness estimation.

**Fig. 4:**
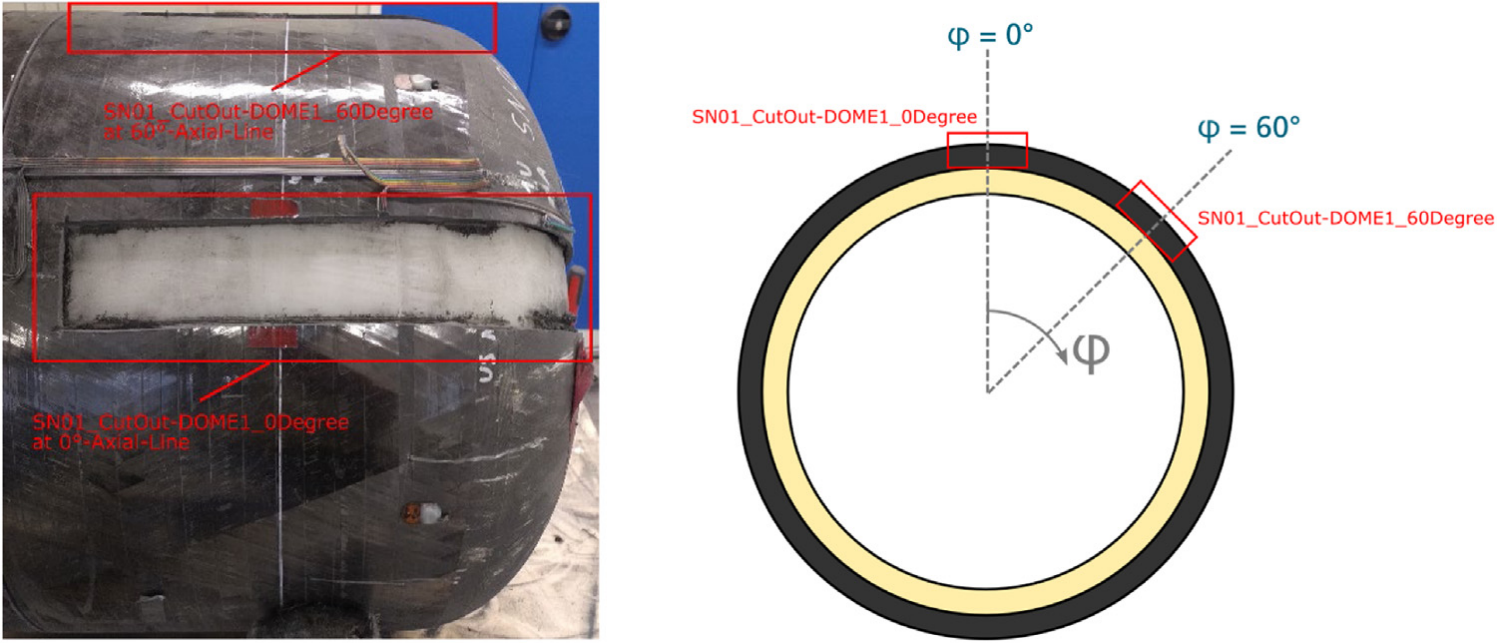
Cut outs from vessel SN01 for thickness measurement.

After manufacturing, vessel SN01 was inspected by structured-light 3D scanning using the measurement system ATOS by Carl Zeiss GOM Metrology GmbH. The evaluation of a best fit cylinder within the cylindrical part yield an outer diameter of 419.87 mm for the manufactured vessel SN01. To obtain further information on the winding thickness realised by the manufacturing process, two cut outs are extracted from Dome 1 of vessel SN01 as shown in Fig. 4. The angle distance between both cut outs is 60°.

**Fig. 5:**
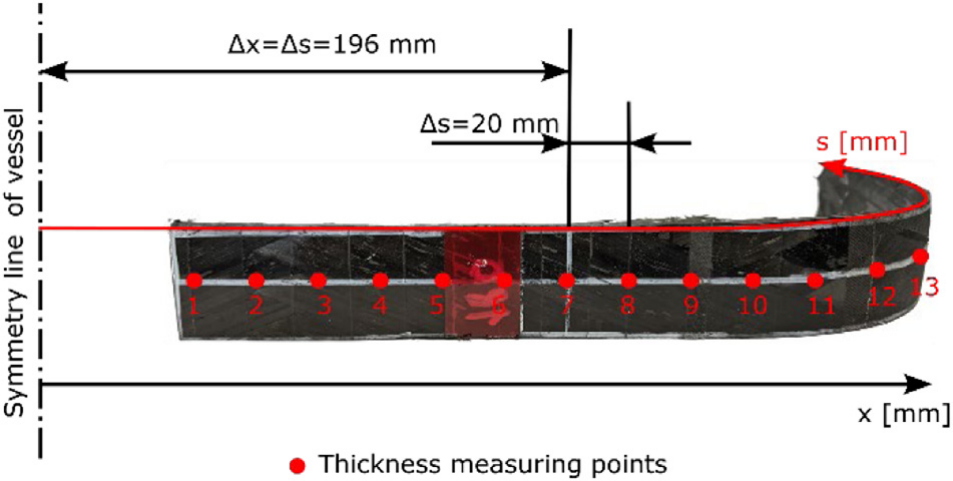
Measuring points on the cut outs of Dome 1 of vessel SN01. Here, s denotes the coordinate along the outer contour and x the axial coordinate. The origin for both coordinates lies on the vessel's symmetry line. On the picture only 13 of the 23 measuring points are marked.

At both cut outs, 23 equally spaced measuring points are defined on the outer surface along the contour coordinate *s*, ref. Fig. 5. The distance *Δs* between the measuring points along the outer contour is 20 mm. At each measuring point, the thickness is measured three times using a micrometer gauge with one flat measuring surface and one spherical measuring surface. From these three values, the mean thickness value and the root mean square deviation is derived for each measuring point.

Based on the thickness values obtained at the measuring points located in the cylindrical regime (measuring points 1 to 10) the mean thickness value of the cylindrical winding section is determined to be 3.28 mm for the cut out at the 0° axial line and 3.30 mm for the cut out at 60° axial line. The detailed results of the thickness measurement are provided in the file “SN01_cutouts_thickness_measurement.xlsx” of the referenced dataset. Please note that at measuring point 14 of cut out SN01_CutOut-DOME1_0Degree the thickness value could not be determined because of a strain gauge applied at this position. The strain gauge at position 7 of SN01_CutOut-DOME1_0Degree could be removed so that the thickness at measuring point 14 could be measured at this cut out.

### 4.2 Pre-test ultrasonic inspection

**Fig. 6:**
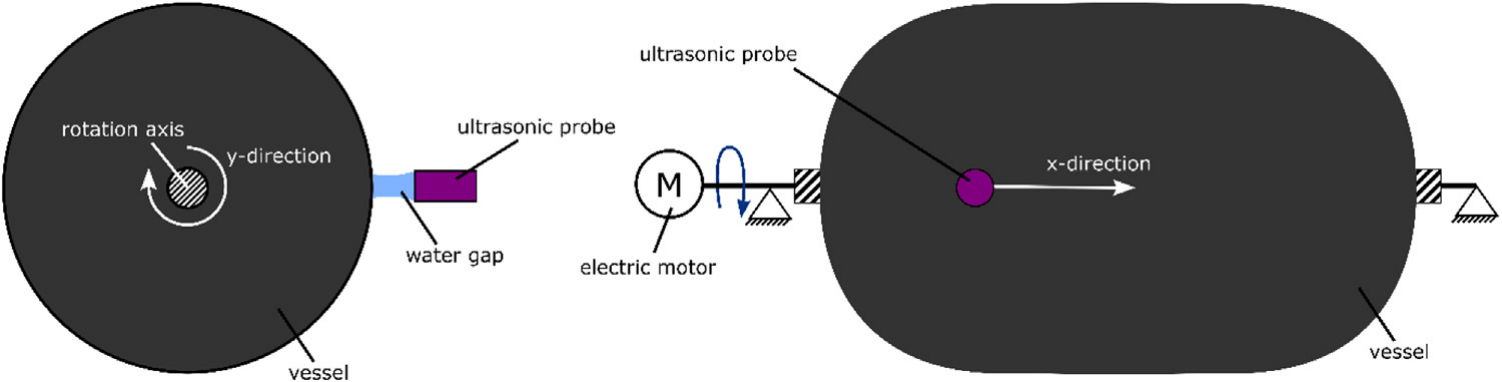
Test setup for ultrasonic inspection.

As depicted in Fig. 6, for ultrasonic inspection the vessel was axially mounted at the bosses allowing to rotate around its longitudinal axis. The scanning was performed with the ultrasonic system Hillger USPC 3040 DAC from the company Hillger NDT GmbH and a 5 Mhz ultrasonic probe. The ultrasonic probe was mounted with a distance of 2 inch to the vessel surface. The ultrasonic signal was coupled into the structure by water jet (squirter technique). While scanning, the sensor moved along the tank axis (x-direction of the scan). When it finished the scan along the axis, the tank was incrementally rotated by an electric motor and the next horizontal line was scanned, allowing the ultrasonic scan to cover the whole cylinder (y-direction of the scan). The dome regimes were not covered by the ultrasonic measurement. The resolution of the ultrasonic scan is 0.5 mm in x-direction and 0.5145 mm in y-direction along the circumference of the tank.

**Fig. 7:**
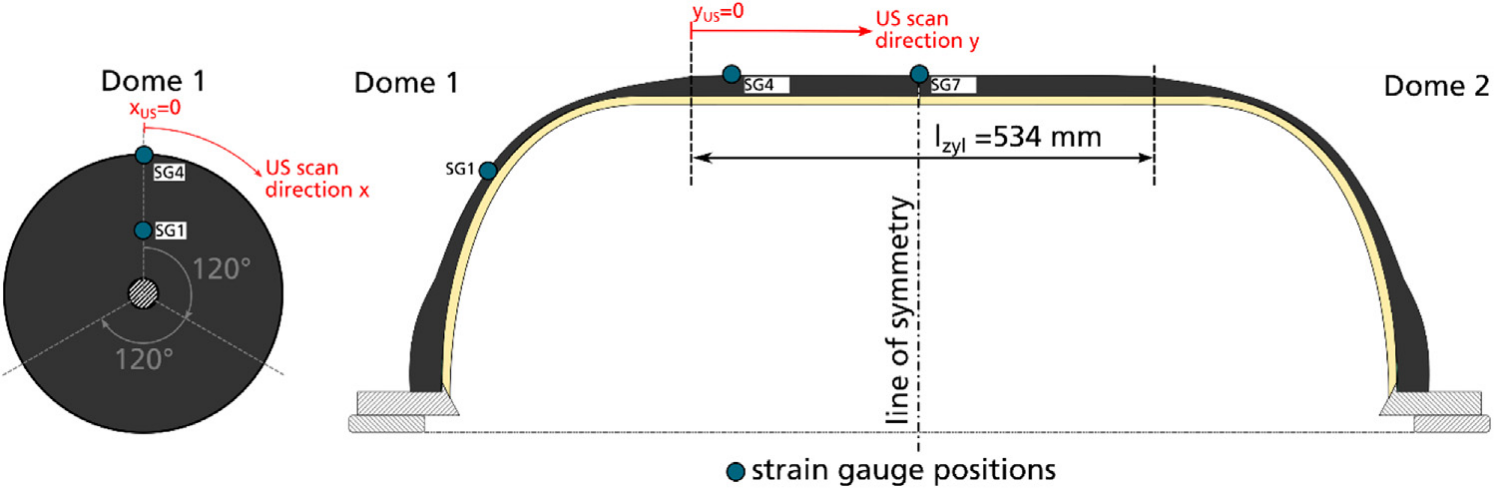
Position of coordinate system of ultrasonic (US) scan.

The origin of the ultrasonic scan coordinate system is located at the transition point from cylinder to dome 1 and at the mandrel line where strain gauge positions 1, 4 and 7 (explained in the next subsection) are located. The ultrasonic inspection has been conducted before the instrumentation of the vessel with strain gauges.

**Fig. 8:**
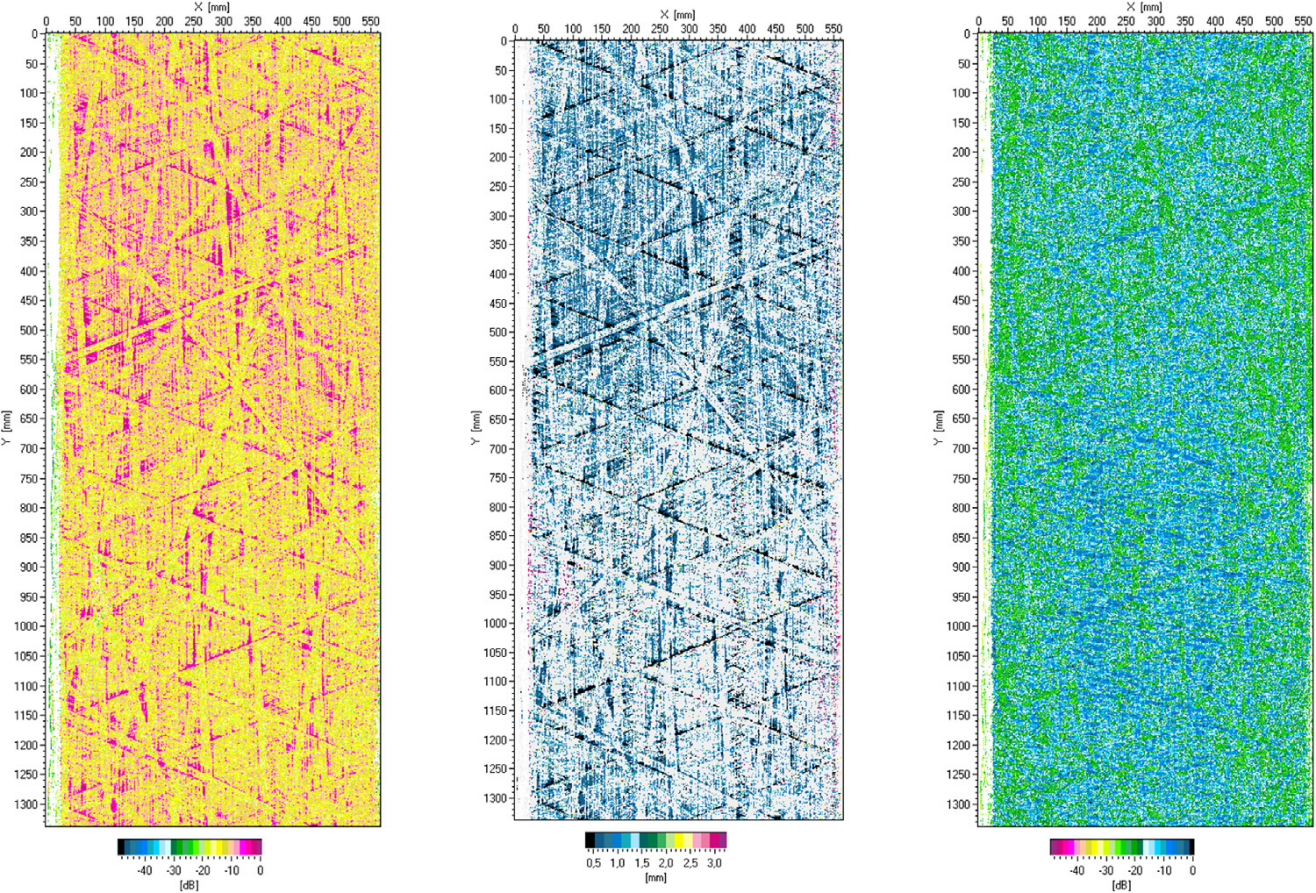
Ultrasonic scan results for cylindrical part of tested vessel SN03: C-Scan of the flaw echo (left), D-Scan providing flaw depth (centre) and C-Scan of the back-wall echo (right).

The ultrasonic measurement provides C- and D-Scans of the cylindrical part of the tested vessel SN03, shown in Fig. 8.

### 4.3 Instrumentation

**Fig. 9:**
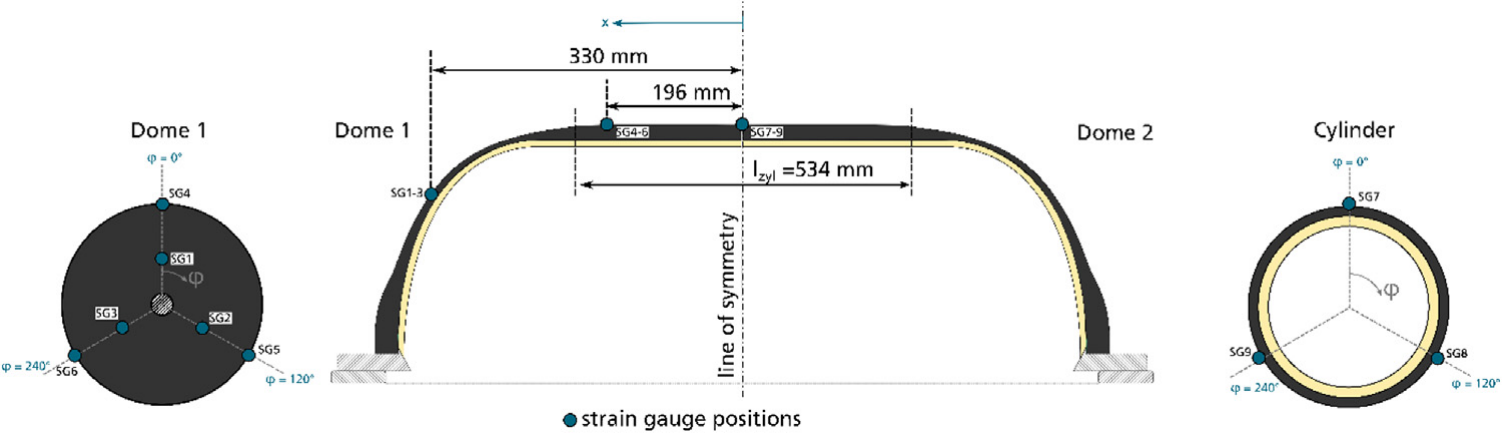
Strain gauge positions; at each position one strain gauge is applied for axial and one for hoop strain measurement; the coordinate system to describe strain gauge positions (x, φ) is also depicted.

To record the mechanical behavior of the vessel SN03 during the burst test, the tank was instrumented with 18 strain gauges at nine positions marked in Fig. 9.

At three axial positions three strain gauge positions were defined along the vessel's circumference with an angle distance of φ=120°. At each strain gauge position, two linear strain gauges (type 1-LY11-6/120 from company HMB) were applied, one measuring the strain in hoop and the other in axial direction. During the test, the strains were recorded with a sample rate of 300 Hz.

### 4.4 Burst pressure test

**Fig. 10:**
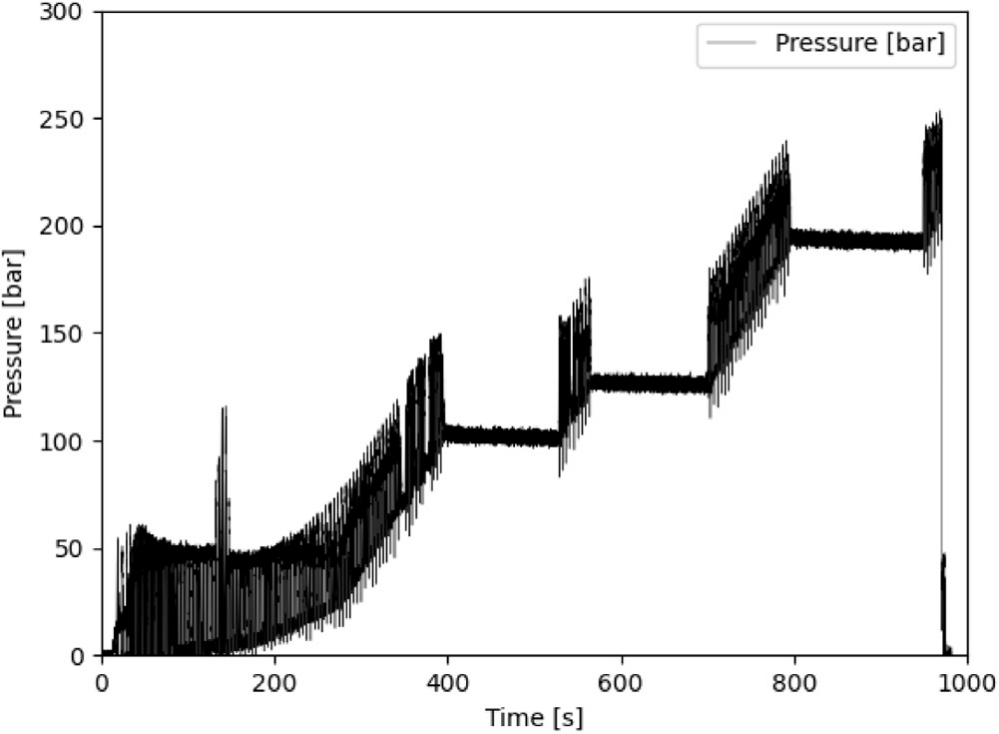
Applied pressure over time sensed by the pressure sensor located near the pump.

The vessel was pressurized by a hydraulic system using water as test fluid. The pressure was applied via a reciprocating piston pump as linear ramp with approximately 2 bar/s. The applied pressure was recorded by a pressure sensor (manufacturer WIKA, Type HP-2-S), located near the pump, with a sample rate of 100 Hz. As shown in Fig. 10, the applied pressure was hold constant at pressure levels (holding intervals) of approximately 100 bar, 125 bar and 200 bar and then further increased after 60 s.

The oscillation of the pressure signal in Fig. 10 is caused by the functional principle of the reciprocating piston pump. When the pump changes from conveying the fluid to intake new fluid, an abrupt pressure increase is recorded at the pressure sensor. Due to the throttling effect of the long (approx. 6 m) fluid pipe to the vessel, the vessel was not exposed to these pressure oscillations. At pressures below 50 bar there is an additional effect causing larger pressure oscillations: Up to this pressure, small air pores within the fluid are compressed allowing a constant increase of the fluid pressure (and thus, internal pressure to the tank) only when all pores are closed.

**Fig. 11:**
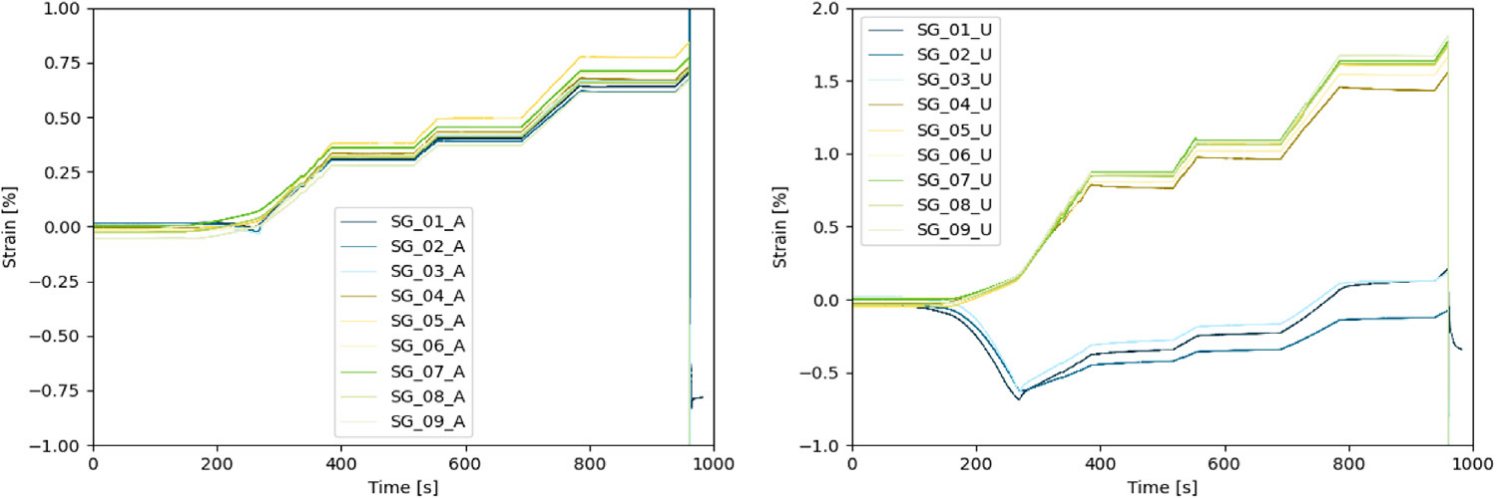
Measured strains over time in axial direction (left) and radial direction (right); strain gauges are grouped by colour: green for strain gauges at position *x* = 0 mm, yellow for strain gauges at position *x* = 196 mm, blue for strain gauges at position *x* = 330 mm.

**Fig. 12:**
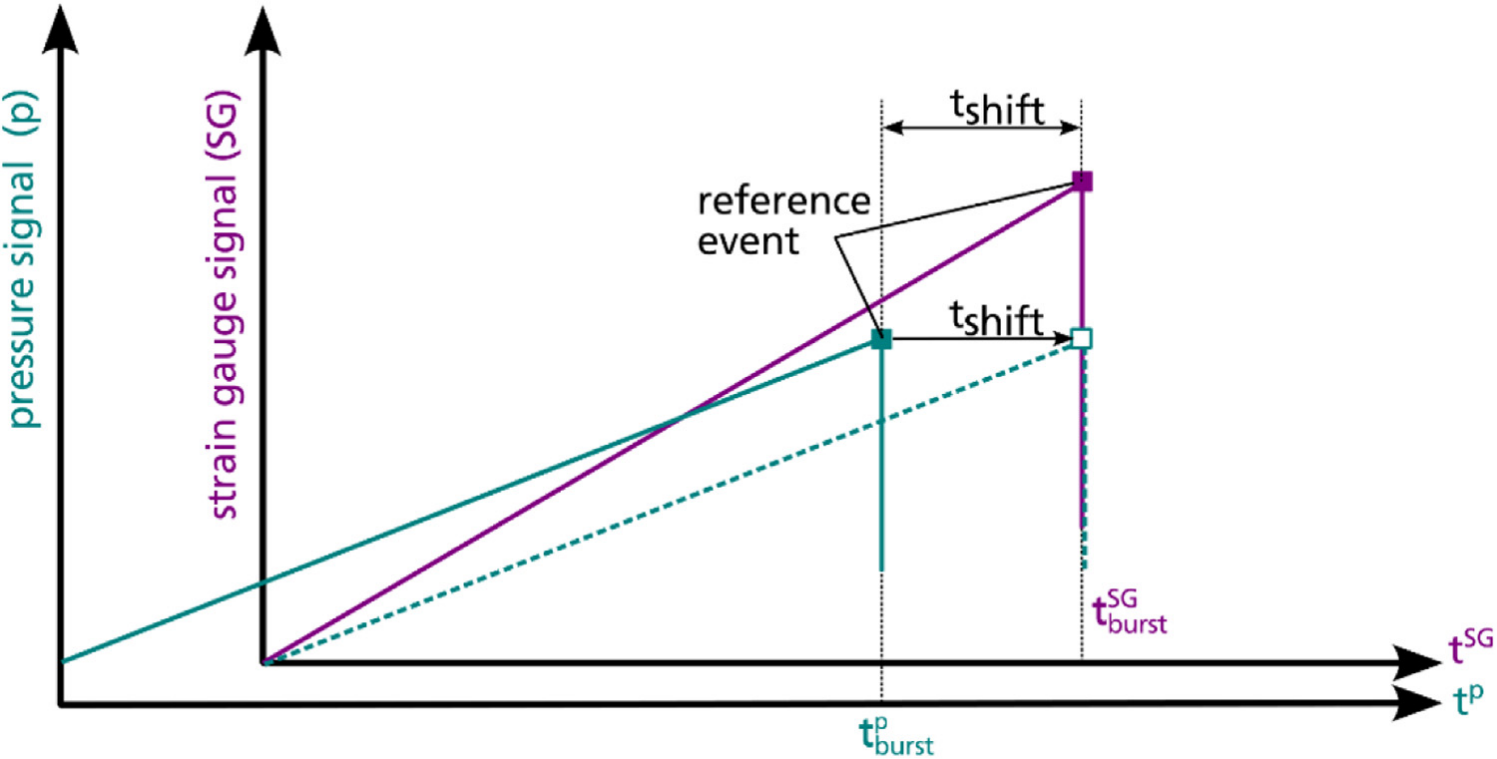
Subsequent signal synchronization by time using burst failure as reference event.

The strain gauge data recorded during the burst pressure test are plotted in Fig. 11. Be aware, that the time signal of the raw pressure data and the raw strain gauge data are not synchronized. Within the dataset a synchronization of both data by time is provided in file “straingauges_and_pressure_10hz_SN03.csv”. The synchronization method illustrated in Fig. 12 makes use of the fact that the gradients of both signals abruptly change at the time of burst failure, that is used as reference event for the synchronization: In both signals, it is searched for the time point where the highest gradient occurs ($t_{burst}^{p}$ for pressure and $t_{burst}^{SG}$ for strain gauge data), indicating burst failure. To synchronize the data, the time difference $t_{shift}$ is added as constant time step to the pressure time signal leading to aligned reference events “burst failure” for both data. For a better handling of the data, the synchronized dataset resolution is reduced to 10 Hz. However, based on the raw data the synchronization could be reproduced for other sample rates.

## Limitations

The limitations related to the data are described below.

*Limitations of geometry measurement*

Geometry measurement of the manufactured vessels is only available for a tank that has not been tested. For the tested vessel the nominal dimensions are provided only. However, both vessels are nominally identical and belong to the same manufacturing batch.

*Limitations of ultrasonic measurement*

The ultrasonic measurement covers the cylindrical part of the tested vessel only. The dome regimes were not inspected. Thus, no information on potential initial defects (e.g. delamination) in the dome regimes are available.

*Limitations of time series data synchronization*

**Fig. 13:**
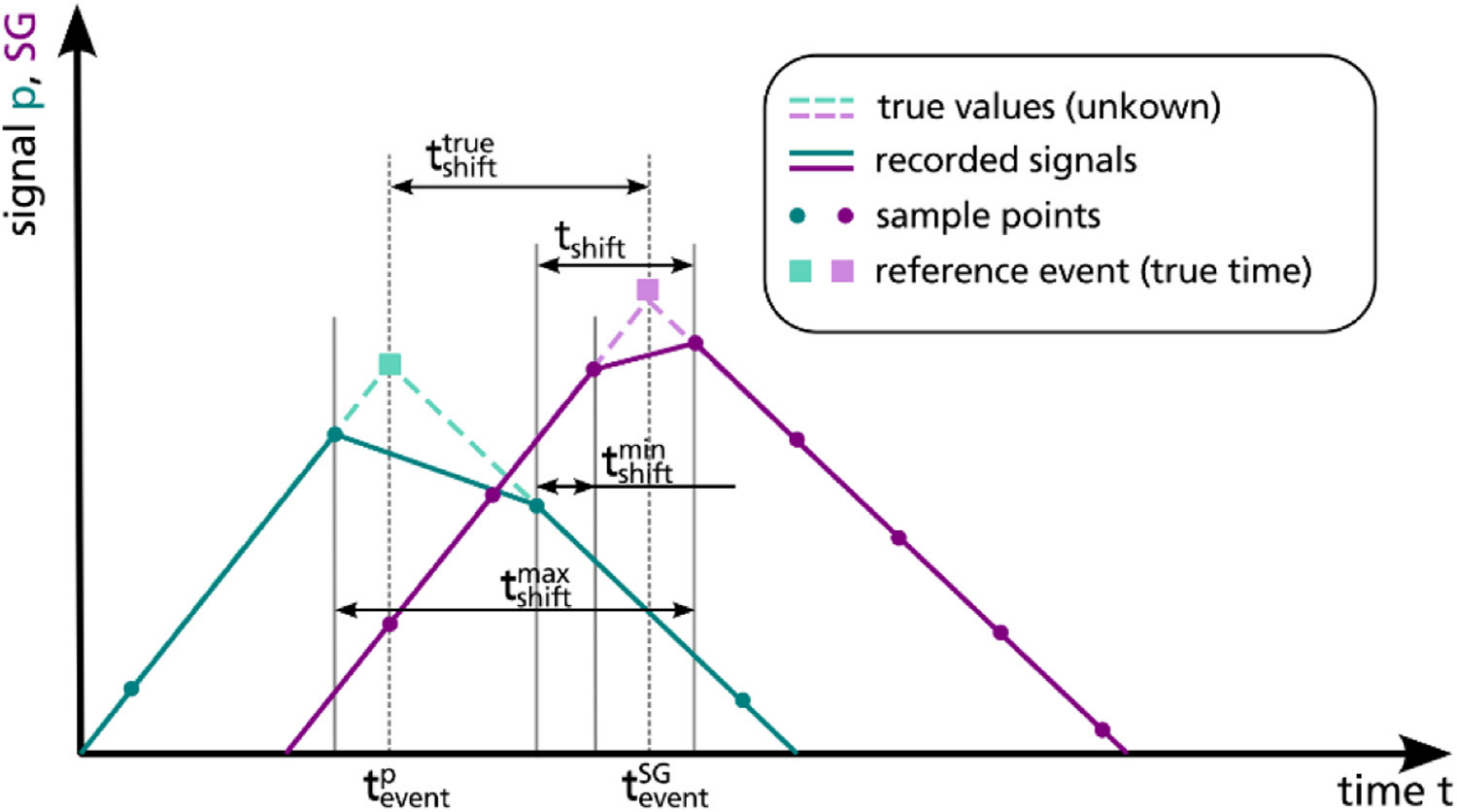
Illustration of uncertainty of subsequent synchronisation by time related to signal sampling.

The raw data of the strain gauge and pressure signals do not refer to the same time signal. This does not limit the value of the data itself but demands for further data processing. The signals can be synchronized by time by choosing a reference event and shifting one signal along the time axis until the reference events are coincident for both signals. Exemplarily, a subsequent synchronization of the data by time is included in the dataset using the burst failure of the vessel as reference event (further explained in section “Burst pressure test”). Alternatively, the holding intervals can be used as reference events, as the pressure signal as well as the strain gauge signals are approximately constant within these intervals. The synchronization procedure based on reference events assumes the physical deformation of the vessel occurring simultaneously to the measured pressure change, that is the pressure measured at the pump is ideally correlated with the measured strain. An additional systematic uncertainty of the synchronization relates to the signal sampling as illustrated in Fig. 13. The reference event most likely is not recorded at its true time point ($t_{event}^{p}$ or $t_{event}^{SG}$, respectively) but data points before and after the event are available only. Thus, the time shift $t_{shift}^{true}$ cannot be calculated exactly but is approximated using the recorded time points framing the reference event. The related uncertainty can be assessed by a variation of the framing recorded time points used to estimate the time shift, e.g. comparing a synchronization using $t_{shift}^{min}$ or $t_{shift}^{max}$ (ref. Fig. 13).

*Limitations of test sample size*

**Fig. 14:**
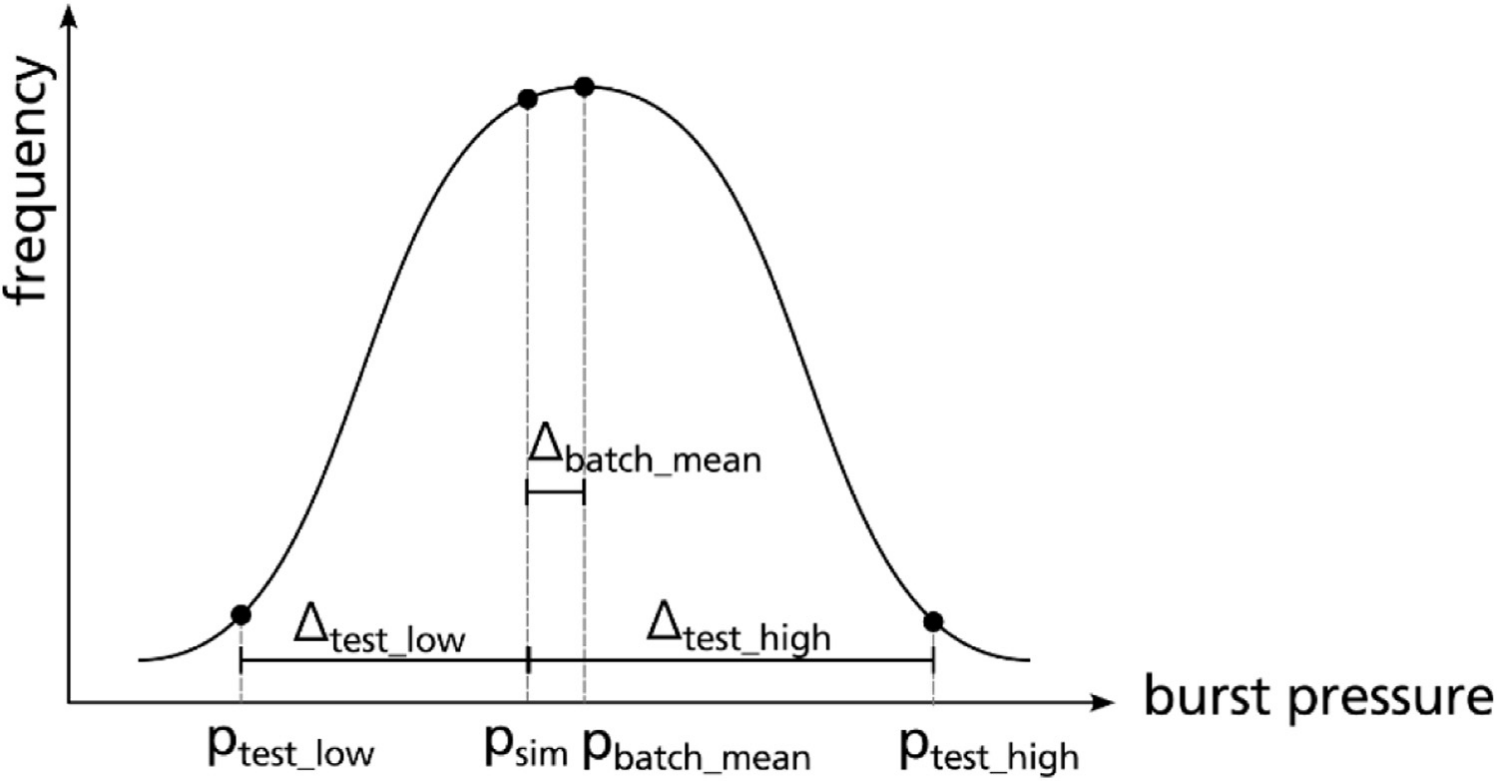
Schematic frequency distribution of burst pressure of a batch of composite vessels.

Only one tank sample was tested and therefore no statistical evaluation is possible. When performing burst pressure tests on multiple composite vessels of a single batch a scattering of the measured burst pressure is expected, schematically shown in Fig. 14. With burst pressure data of several tank samples available, mean value ($p_{batch\_mean}$), standard deviation and a confidence interval for the mean value could be estimated. Burst tests by Nebe [2] reveal a standard deviation up to 4.3 % depending on the laminate's stacking sequence. The Regulation No 134 [3] used for composite pressure vessel certification allows for a scatter of ±10 % around the burst pressure mean value. As only one sample was tested, statistical evaluation is not possible for the referenced dataset. When comparing the burst pressure of another vessel design to the present one, comparison is only possible to the individual burst pressure of the specific tested vessel. The average burst pressure $p_{batch\_mean}$ belonging to the present vessel design is unknown. Furthermore, it is unknown if the tested vessel provides a comparatively low (e.g. $p_{test\_low}$) or comparatively high (e.g. $p_{test\_high}$) burst pressure with respect to the (unknown) mean burst pressure $p_{batch\_mean}\;$of the batch. This should be kept in mind when using the dataset for validation of burst pressure analyses as illustrated in Fig. 14. Here, a burst pressure analysis is assumed that predict the burst pressure ($p_{sim}$) slightly (by $\Delta_{batch\_mean}$) below the (unknown) mean burst pressure of the batch $p_{batch\_mean}.$ When comparing the predicted burst pressure $p_{sim}$ to the burst pressure of the single tested vessel, two extreme scenarios may occur:

(1) The burst pressure of the tested vessel is comparatively low ($p_{test\_low}$) and the simulation seems to overestimate the burst pressure by $\Delta_{test\_low}$ and could be judged to be not conservative also it is conservative with respect to the (unknown) mean burst pressure of the batch.
(2) The burst pressure of the tested vessel is comparatively high ($p_{test\_high}$) and the simulation seems to underestimate the burst pressure by $\Delta_{test\_high}$.

To conclude, the underlying sample size of the referenced dataset is too small to finally quantify the error of a burst pressure prediction. Nevertheless, the dataset is useful to get an estimation of the model accuracy and to get insight into the mechanical behaviour of composite pressure vessels. Furthermore, it can provide a use case for comparing different burst pressure simulation approaches.

## Ethics Statement

The authors have read and follow the ethical requirements for publication in Data in Brief and confirm that the current work does not involve human subjects, animal experiments, or any data collected from social media platforms.

## Credit Author Statement

**Caroline Lüders:** Conceptualization, Methodology, Investigation, Writing - Original Draft. **Sven Ropte:** Conceptualization, Methodology, Investigation, Writing - Review & Editing. **Daniel Schmidt:** Conceptualization, Methodology, Investigation, Writing - Review & Editing. **Martin Liebisch:** Formal analysis, Data Curation, Writing - Review & Editing, Visualization.

## Data Availability

zenodoHydraulic burst pressure test of Type IV composite pressure vessel (Original data).
